# Metastable orientational order of colloidal discoids

**DOI:** 10.1038/ncomms9507

**Published:** 2015-10-07

**Authors:** Lilian C. Hsiao, Benjamin A. Schultz, Jens Glaser, Michael Engel, Megan E. Szakasits, Sharon C. Glotzer, Michael J. Solomon

**Affiliations:** 1 Department of Chemical Engineering, University of Michigan, Ann Arbor, Michigan 48109, USA; 2 Department of Physics, University of Michigan, Ann Arbor, Michigan 48109, USA; 3 Department of Materials Science and Engineering, University of Michigan, Ann Arbor, Michigan 48109, USA; 5 Present address: Department of Chemical Engineering, Massachusetts Institute of Technology, Cambridge, Massachusetts 02139, USA

## Abstract

The interplay between phase separation and kinetic arrest is important in supramolecular self-assembly, but their effects on emergent orientational order are not well understood when anisotropic building blocks are used. Contrary to the typical progression from disorder to order in isotropic systems, here we report that colloidal oblate discoids initially self-assemble into short, metastable strands with orientational order—regardless of the final structure. The model discoids are suspended in a refractive index and density-matched solvent. Then, we use confocal microscopy experiments and Monte Carlo simulations spanning a broad range of volume fractions and attraction strengths to show that disordered clusters form near coexistence boundaries, whereas oriented strands persist with strong attractions. We rationalize this unusual observation in light of the interaction anisotropy imparted by the discoids. These findings may guide self-assembly for anisotropic systems in which orientational order is desired, such as when tailored mechanical properties are sought.

## Abstract (web summary)

The pathways available for self-assembly are affected by the shape anisotropy of the building blocks, but the details are still unclear. Here, Hsiao *et al*. show that colloidal discoids self-assemble into metastable states with orientational order when kinetic trapping is incorporated as a design principle.

## Graphical abstract

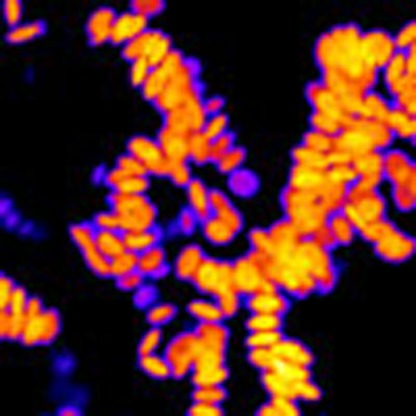

Proteins, microtubules and DNA are examples of molecules that self-assemble into ordered strands under conditions found in nature^1^,^2^,^3^. These natural architectures, such as chiral helices and bundles, have inspired the development of their synthetic counterparts through the use of external fields^4^,^5^,^6^, surface modification^7^ and confinement^8^. The combination of shape and interaction anisotropies used in these assembly methods has a direct impact on the availability of kinetic pathways and the degree to which orientational order may be achieved. Recent simulations have shown that the oblate spheroid (discoid) is a promising candidate for the self-assembly of strands, in which discoids are orientationally aligned with face-to-face (F–F) contacts^9^,^10^. This can potentially be accomplished without the need for complex surface modification: minimization of the free energy predicts that the tilted stack configuration can be favourable for discoids at finite temperatures, resulting in twisted and columnar structures with long-range orientational order^9^,^11^.

Controlling the relative range of the attractive interaction, *ξ*, is essential to successful oriented assembly. The use of short-range attractions is undesirable because they generally result in disordered assembly^12^. Longer interaction ranges generally result in a greater degree of freedom, provided that particles remain within the attractive well^13^. The kinetics of self-assembly play an important role in determining the type of structure formed, even with isotropic spheres. For *ξ*⩾0.3 and shallow quenches, liquid–gas phase separation proceeds rapidly and yields compact clusters and spinodal-like networks^14^,^15^. When the system is quenched by increasing the attraction strength, non-equilibrium structures form deep within the region of phase instability. In this region, local minima in the free energy landscape give rise to kinetically trapped, metastable structures like gels^16^. Quenching can be accomplished experimentally by raising the concentration of non-adsorbing molecules in depletion-driven systems^17^, by lowering the temperature of thermoreversible adhesive hard spheres^18^,^19^ or by increasing the extent of interdroplet bridging in thermogelling nanoemulsions^20^,^21^.

When shape anisotropy is added to the competition between energetics and kinetics, the driving force behind self-assembly becomes less clear. Here, metastable states not found with isotropic spheres can give rise to unusual self-assembled structures. Confocal microscopy is an effective tool to study colloidal assembly with anisotropic particle shapes^4^,^22^,^23^,^24^,^25^. Thus far, significant effort has been made to study discoidal assembly using suspensions of clay platelets. These colloids show unique phase behaviour such as empty liquids^26^, but their size is typically on the order of nanometers—too small for direct imaging. Existing methods used to generate colloidal polymeric discoids in limited quantities include biaxial stretching^27^ and blown films^28^.

We use uniaxial compression to generate model colloidal discoids that can be clearly visualized in three dimensions (3D) without complications from sedimentation. Depletion interactions between the colloids and non-adsorbing polymer molecules (spherical depletants) are then used to drive assembly. We demonstrate that the precise control of dynamical arrest is key to achieving different types of microstructure with colloidal discoids. Experiments and simulations, spanning a wide range of colloidal volume fractions (10^−3^≤*φ*≤10^−1^) and attraction strengths (3≤*U*_depl_/*kT*≤84, estimated according to the F–F configuration of discoids), show that long strands with orientational order persist when the attraction is strong. Here, *k* is the Boltzmann constant and *T* is the temperature. Phase separation becomes rapid at weaker attraction strengths and produces disordered clusters. An unexpected result is that the initial stages of discoidal assembly are dominated by the formation of metastable aligned contacts of discoids, regardless of the final structure and the attraction strength. We provide an explanation for this observation that rests on the interaction anisotropy imparted by the discoidal shape, and discuss the effect of the competition between energetics and entropy on the assembly structure and kinetics. Because of the simplicity of the interaction potential and the shape anisotropy of our model system, these findings can be used to broadly understand and guide the self-assembly of anisotropic building blocks^29^,^30^ into one-dimensional (1D) structures.

## Results

### Phase behaviour and microstructure of oriented assembly

**Figure 1:**
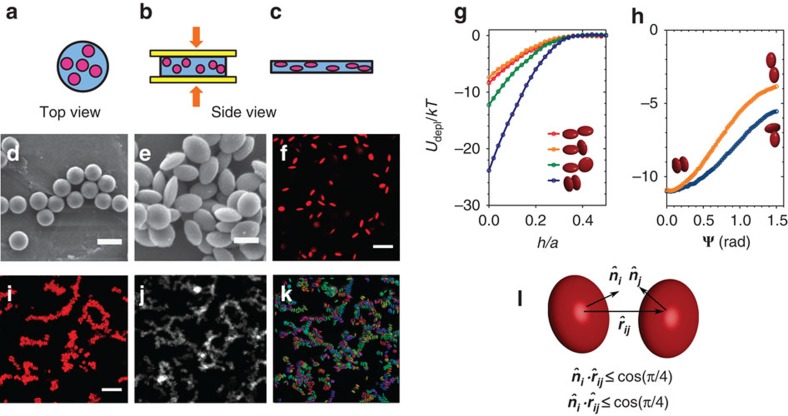
Self-assembly of discoids and image processing. Monodisperse PMMA discoids are generated by (**a**) embedding PMMA spheres in a PDMS matrix, (**b**) applying a uniaxial compression at temperatures *T*>*T*_g_, and (**c**) cooling under pressure. (**d**) Representative SEM image (scale bars, 2 μm) of the precursor PMMA spheres and (**e**) of the discoids. (**f**) Representative confocal microscopy image of a dilute suspension (volume fraction *φ*=0.01) of stable discoids (scale bar, 15 μm). (**g**) Anisotropic depletion potential of discoids (concentration *c*/c*=1.5) in edge-to-edge (red), edge × edge (orange), edge-to-face (green) and face-to-face (blue) configurations. (**h**) Depletion potential of discoids as a function of their relative orientation Ψ (see text). Performance of the watershed-cut image processing is demonstrated using: (**i**) a raw 3D confocal image (projection from a Δ*z*=10 μm volume) for a sample at *φ*=0.02, *c*/c*=4.0 (scale bar, 10 μm); (**j**) same image after thresholding; (**k**) overlay of the identified discoids on the thresholded image. (**l**) Definition of the orientational criterion between two discoids.

The colloids used in this study are monodisperse, sterically stabilized discoids with aspect ratio, *l*=*b*/*a*=(0.46±0.09), obtained using the method of thermomechanical compression (Fig. 1a–c, Methods). The batch synthesis provides ∼10^−1^ g of material after purification. Here, *a* is the radius of the major axis and *b* is the radius of the minor axis; for the discoids, *a*=(1.07±0.06) μm and *b*=(0.49±0.08) μm. Figure 1d,e shows representative scanning electron microscopy (SEM) images of the precursor spheres and the final discoids. The discoids are stable in suspension for days (Fig. 1f, Methods, Supplementary Fig. 1). They are suspended in a refractive index and density-matched solvent at volume fractions varying from *φ*=0.003 to 0.04 to allow for 3D structural characterization using confocal microscopy. Self-assembly is induced by addition of monodisperse polystyrene at various concentrations (1.1≤*c*/c*≤5.3, where c* is the overlap concentration of the polystyrene) to the suspension. The polystyrene molecule acts as a depletant and generates a long-range attraction (polymer-colloid size ratio, *ξ*_a_=*R*_g_/*a*=0.18, *ξ*_b_=*R*_g_/*b*=0.40). In addition, we use Monte Carlo (MC) simulations to investigate the self-assembly of the discoids for a range of volume fractions and depletant concentrations (*φ*=0.01, 0.02, 0.05, 0.10 and 0.2≤*c*/c*≤4.0) that spans the experimental conditions. The discoids and ideal polymer depletants experience volume exclusion effects, but the polymers are mutually penetrable^31^. To improve the speed of the MC simulations, we use an implicit treatment of depletants in the overlap regions around the colloids^32^. Further details of the simulation method are available in Methods and Supplementary Notes 1 and 2.

Discoids energetically favour the F–F configuration. We directly calculate a depletion potential, *U*_depl_, generalizing the Asakura–Oosawa potential^33^ from the free volume available to mutually penetrable, ideal depletants (Fig. 1g,h). The depletion potential as a function of the discoid surface separation, *h*, is at a maximum when Ψ=0. Here, Ψ is defined as the angular difference between the centre-to-centre vector (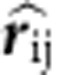,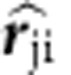) and the normal vectors (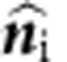,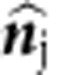) of the discoids.

From the confocal microscopy image volumes, we compute the volume fraction, positions and orientations of the discoids with a watershed-cut-based image processing algorithm that has been previously applied to granular materials^34^. This method can be used to characterize discoids with different values of *l* within the same image. Figure 1i–k shows that the intensity-based algorithm is accurate in identifying the position and orientation of discoids in close contact. The algorithm and its error characterization are presented in the Methods section.

Particles are considered bonded (clustered) when they are within surface separation distances equivalent to the first minimum of the orientationally dependent radial surface distribution function (for example, particles are in contact at *h*≤0.55 μm for the case of *c*/c*=4.0, *φ*=0.02). We define the fraction of particles in these aggregates (where an aggregate is a cluster of size *s*⩾3) as *f*_clust_, where large values of *f*_clust_ indicate a significant subpopulation of particles residing within clusters. Orientational order between two discoids is quantified using an angular criterion (Fig. 1l), 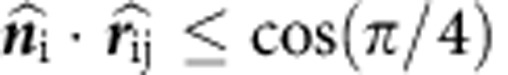 and 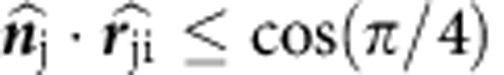. This criterion yields the fraction *f*_ordered_ of particles in oriented clusters with *s*⩾3. Assemblies with strong attractions tend to have a significant subpopulation of particles in F–F configurations and thus have high values of *f*_ordered_ (Supplementary Fig. 2).

**Figure 2:**
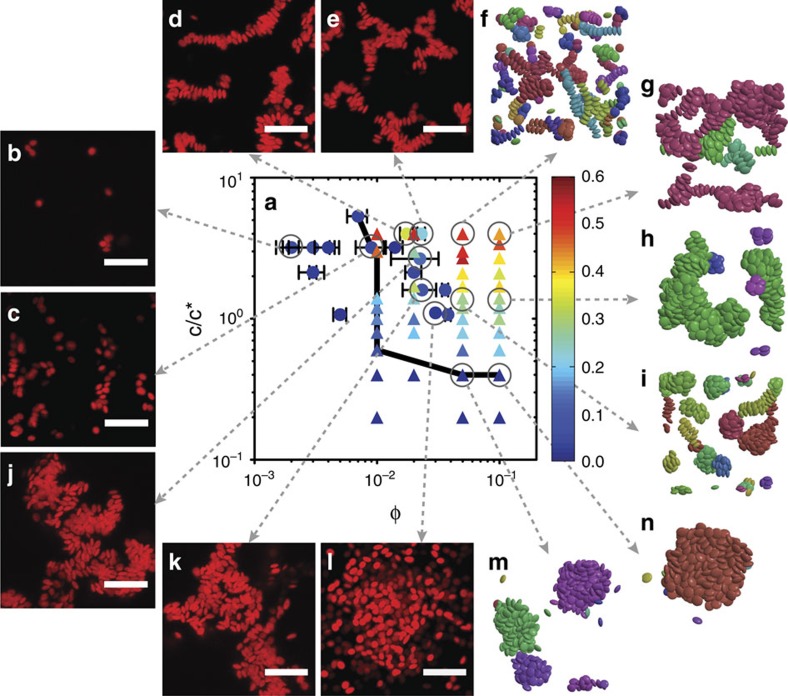
Phase behaviour of discoids as a function of attraction strength and volume fraction. (**a**) Phase diagram with experimental (circles) and simulation (triangles) state points. The colour indicates the value of *f*_ordered_ (inset). Solid black line demarcates coexistence boundaries. Error bars represent s.e.m., *n*=3. (**b**–**n**) Representative confocal microscopy images and simulation renderings of state points. Scale bars, 10 μm. Particles are coloured by cluster in simulation snapshots.

Figure 2a illustrates the effect of *c*/c* and *φ* on the degree of orientational ordering of colloidal discoids (*f*_ordered_) in both experiments and simulations. All experimental data are reported at a waiting time of *t*_w_=120 min, and simulations are reported for number of sweeps, *t*_MC_, between 40 and 500 million. The coexistence boundary (solid line in Fig. 2a) is defined when *f*_clust_>0.2 (a phase diagram with *f*_clust_ is in Supplementary Fig. 3). Samples to the left of the solid line at low *φ* exist as suspensions of mobile free particles or small clusters (Fig. 2b,c), in which little to no ordering can be observed. Samples to the right or on the coexistence boundary are large aggregates. At the highest *c*/c* and *φ* (*c*/c*⩾2.7, *φ*⩾0.02) within the phase diagram, we find strands with high orientational order in both experiments and simulations (0.2≤*f*_ordered_≤0.6), shown in Fig. 2d–g. At intermediate *c*/c* (1.0≤*c*/c*≤2.7), the self-assembled structures become increasingly disordered (Fig. 2h–n). This type of heterogeneous structure interspersed with large voids and regions of dense packing is reminiscent of gels formed with weak, short-range attractions^35^,^36^. The degree of orientational order continues to decrease gradually as *c*/c* decreases, reaching *f*_ordered_<0.1 at the phase boundary. Structures at the phase boundary contain large condensates/clusters that coexist with mobile particles (Fig. 2l–n).

**Figure 3:**
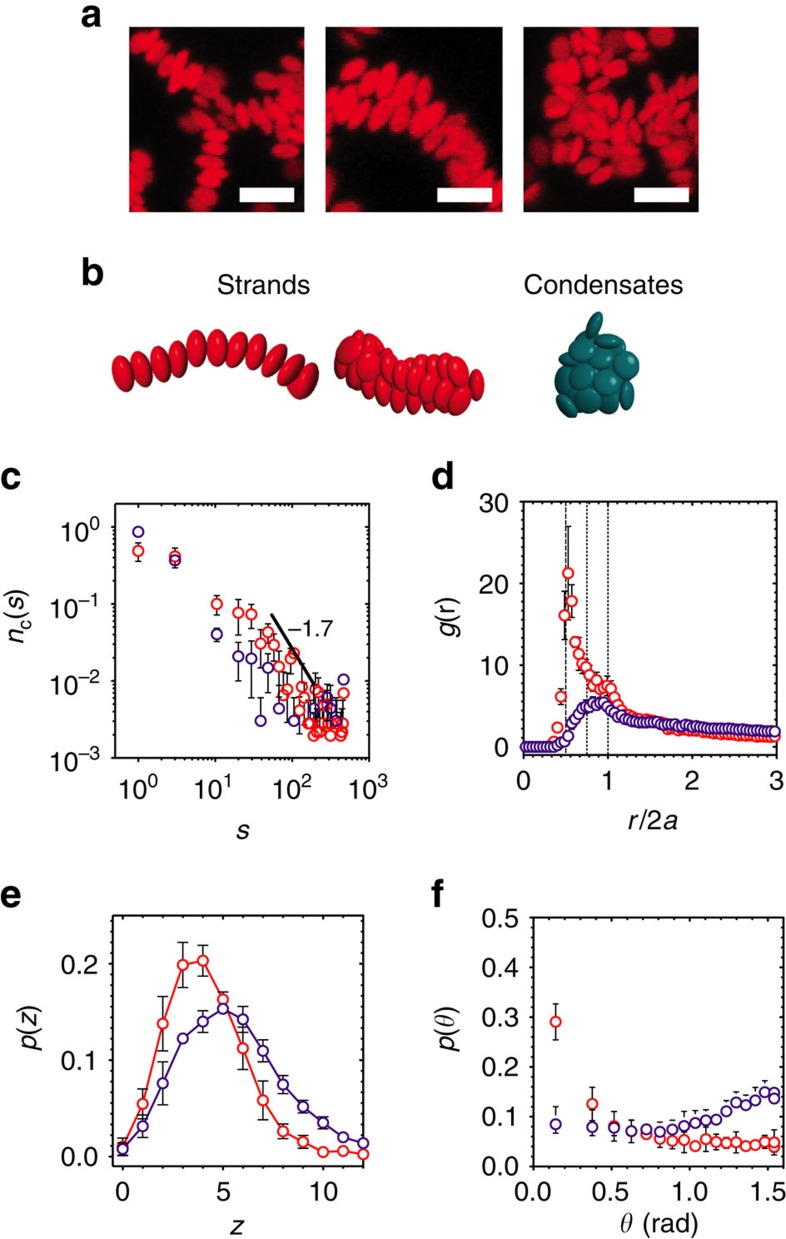
Structural characterization of discoidal assembly. (**a**) Close-up confocal microscopy images (projection from Δ*z*=10 μm volume) of single strands, double strands and disordered clusters. Strands are selected from samples at *c*/c*=4.0, *φ*=0.02 and condensates from *c*/c*=1.7, *φ*=0.02. Scale bars, 3 μm. (**b**) Snapshots of equivalent strands and condensates from simulations. We plot (**c**) the cluster size distribution, (**d**) the radial distribution function, (**e**) the contact number distribution and (**f**) the bond angle distributions for experiments at *c*/c*=4.0, *φ*=0.02 (open red) and *c*/c*=1.7, *φ*=0.02 (open purple) at *t*_w_=120 min. In **c**, the solid line indicates a power law fitting and its corresponding exponent for samples at *c*/c*=4.0, *φ*=0.02. In **d**, dashed, dashed–dotted and dotted lines represent the face-to-face (F–F), edge-to-face (E–F) and edge-to-edge (E–E) configurations. Error bars represent s.e.m., *n*=3.

The visual differences between ordered samples (*f*_ordered_⩾0.2) and disordered samples (*f*_ordered_<0.2) are striking (Fig. 3a,b). Single and double strands are found frequently at high *c*/c* and sometimes contain short helical segments. On the other hand, discoids assemble into condensates with no orientational order at low *c*/c*. We use a number of structural measures to characterize our samples. The cluster size distribution, *n*_c_(*s*), obeys a power law for high *c*/c* samples (Fig. 3c). An exponent of −1.7±0.4 shows that stronger attractions lead to structures that are open and fractal in nature. The radial distribution function *g*(*r*), the contact number distribution *p*(*z*), and the bond angle distribution *p*(*θ*) are used to distinguish strands from condensates (Fig. 3d–f). The value of *g*(*r*) represents the orientationally averaged, density-normalized probability of finding another particle at a distance *r* from the centre of a particle. Noting that the aspect ratio of the discoids is close to 0.5, Figure 3d shows a sharp peak in *g*(*r*) at *r*/2*a=*0.5 for high *c*/c* assemblies, indicating once more that a large subpopulation of the particles exist in F–F configurations. A small secondary peak is seen at *r*/2*a*=1.0, indicative of the small subpopulation of particles existing in edge-to-edge (E–E) configurations and possibly in the secondary coordination shell of the F–F configuration. In contrast, samples at low *c*/c* lack a preference for F–F bonding. Instead, their structures are reminiscent of amorphous materials, with broad, shallow peaks at *r*/2*a*∼0.75 corresponding to the edge-to-face (E–F) configuration.

Because of the tendency of strands to be in the F–F configuration, they show a narrower contact number distribution and a lower mean contact number (<*z*>=3.2±0.9 at *c*/c*=4.0) than disordered structures (<*z*>=4.9±0.7 at *c*/c*=1.7; Fig. 3e). Although there are many single strands with *z*=2 in high *c*/c* assemblies, the microstructure also consists of interconnected and double strands with *z*>2. In the simulations, the strand connectivity, strand diameter, and the average value of *f*_ordered_ in the metastable state depend more strongly on aggregation kinetics at high *c*/c* rather than at low *c*/c* (Supplementary Notes 2 and Supplementary Figs 4 and 5). The strand-like nature is also clear from the bond angle distribution (Fig. 3f). The bond angle is computed from the dot product 
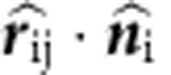
,
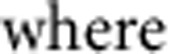
 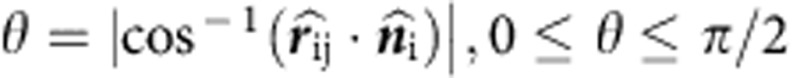. This parameter has been previously applied to characterize strands found in nanoparticle step-growth assembly^37^. The likelihood of finding discoids in strands is *p*(*θ*=0)=0.29±0.04 for high *c*/c* samples, versus 0.08±0.03 for low *c*/c* samples.

### Kinetics of self-assembly

We demonstrated in Fig. 2 that highly ordered structures are found deep in the phase diagram, far from the coexistence boundaries. Our explanation for this observation is the kinetic arrest of phase separation when the system is deeply quenched with strong attractions. We now provide support for this rationale by comparing the growth kinetics of assemblies with high and low orientational order.

**Figure 4:**
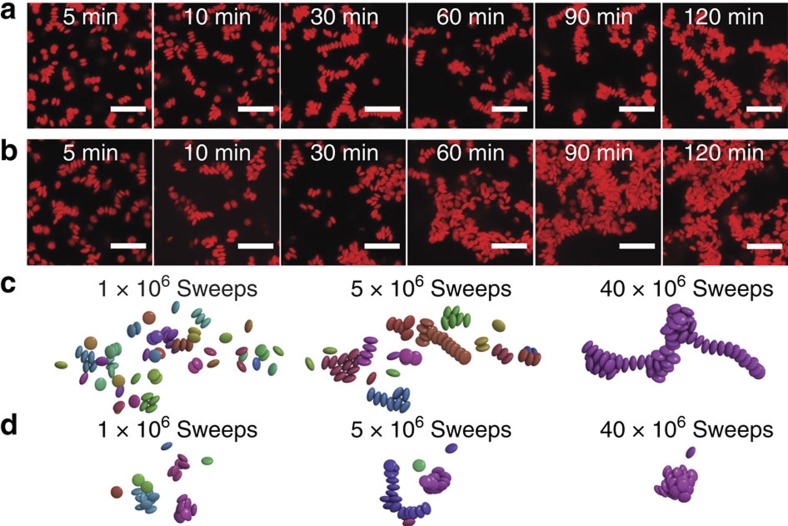
Visualization of the growth in discoidal assemblies. Representative confocal microscopy images (projection from Δ*z*=10 μm volume) of *φ*=0.02 structures at (**a**) *c*/c*=4.0 (characteristic timescales *τ*_clust_=14.1 min, *τ*_ordered_=16.7 min) and (**b**) *c*/c*=1.7 (*τ*_clust_=9.1 min, *τ*_ordered_=24.2 min). Scale bars, 10 μm. Snapshots of simulation structures are for *φ*=0.02 structures at (**c**) *c*/c*=4.0 (*τ*_clust_=5.5 × 10^6^ sweeps, *τ*_ordered_=7.9 × 10^6^ sweeps) and (**d**) *c*/c*=1.7 (*τ*_clust_=0.7 × 10^6^ sweeps, *τ*_ordered_=9.8 × 10^6^ sweeps). Particles are coloured by clusters.

Figure 4a shows the visual evolution of structures with high orientational order (*f*_ordered_=0.36±0.07). At a wait time of *t*_w_=5 min, the system is comprised of mostly free, mobile colloids along with short oriented strands. Between *t*_w_=10 min and 30 min, there is a rapid increase in the population of oriented clusters, which then interconnect to form larger structures. These structures continue to grow until long strands are formed at *t*_w_=120 min. In contrast, Fig. 4b shows that orientational disorder develops relatively early within the system of low orientational order (*f*_ordered_=0.07±0.03). Although short strands are still seen at *t*_w_=5 min, aggregates with little orientational order are formed by *t*_w_=30 min. From this point onwards until *t*_w_=120 min, the clusters grow continuously until heterogeneous regions of dense clusters and large voids are formed. Structures from MC simulations are seen to follow the same trends observed in experiments (Fig. 4c,d). We note that the use of single-particle moves instead of cluster moves^36^ allows us to semi-quantitatively track the kinetic arrest.

**Figure 5:**
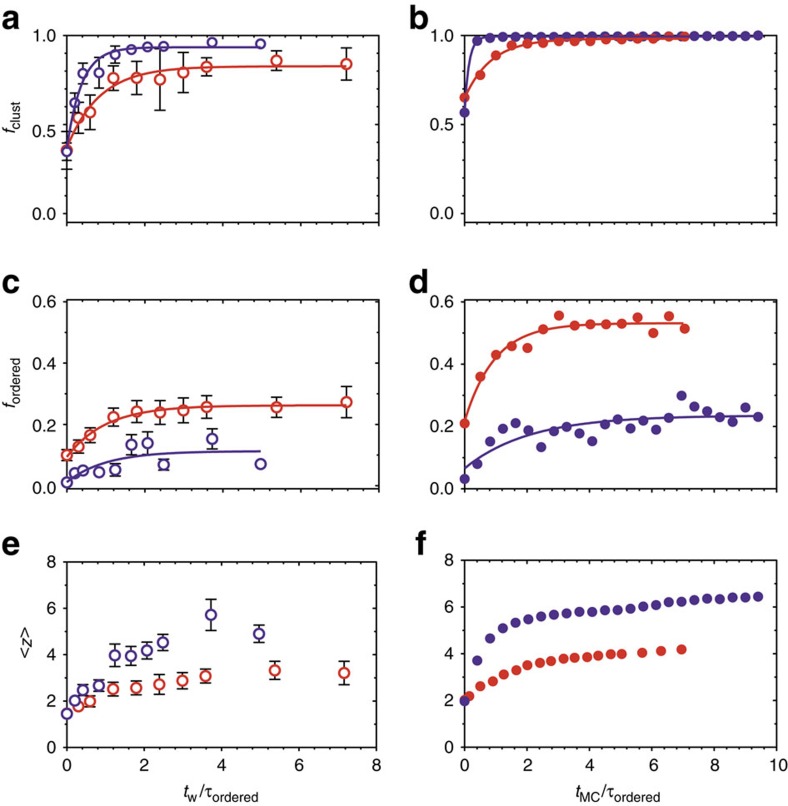
Kinetics of discoidal assembly. (**a**,**c**,**e**) are from experiments and (**b**,**d**,**f**) are from simulations. (**a**,**b**) The fraction of particles in clusters, (**c**,**d**) the fraction of particles in ordered clusters and (**e**,**f**) the mean contact number are plotted as a function of time. To enable qualitative comparison of kinetics, we normalize the time by the characteristic timescale for which orientational order develops, *τ*_ordered_. Error bars represent s.e.m., *n*=3. All data points are shown for *c*/c*=4.0, *φ*=0.05 (red) and *c*/c*=1.7, *φ*=0.05 (purple). Solid lines in **a**–**d** are fits to the kinetics data (see text).

We quantify these structural changes by studying the temporal evolution of *g*(*r*) (Supplementary Notes 3 and Supplementary Fig. 6), *f*_clust_, *f*_ordered_ and <*z*> (Fig. 5). Cluster growth and the development of orientational order occur on different timescales, which we define as *τ*_clust_ and *τ*_ordered_. The timescales are determined from an exponential fitting of the form 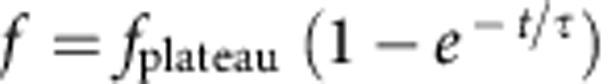 , where *f* refers to *f*_clust_ or *f*_ordered_, and *τ* refers to *τ*_clust_ or *τ*_ordered_. The time *t* refers to *t*_w_ or *t*_MC_, and we use this fitting to obtain the plateau value *f*_plateau_ and *τ*. We normalize the experimental and simulation times by their respective *τ*_ordered_ to provide qualitative comparison of their kinetics.

The kinetics data in Fig. 5 show that orientational order develops more slowly than clustering, particularly with weak attractions. Discoids first assemble into short, metastable strands. For shallow quenches, these condense rapidly into disordered aggregates, but discoids continue to undergo structural rearrangement long after clustering is complete. With stronger attractions, the initial strands interconnect to form longer strands that are kinetically trapped and coarsen slowly over long periods of time. The fact that the observed trends are identical in experiments and simulations, despite differences in the ways particles move, point to the generality of these findings.

### Discoids progress from ordered to disordered states

**Figure 6:**
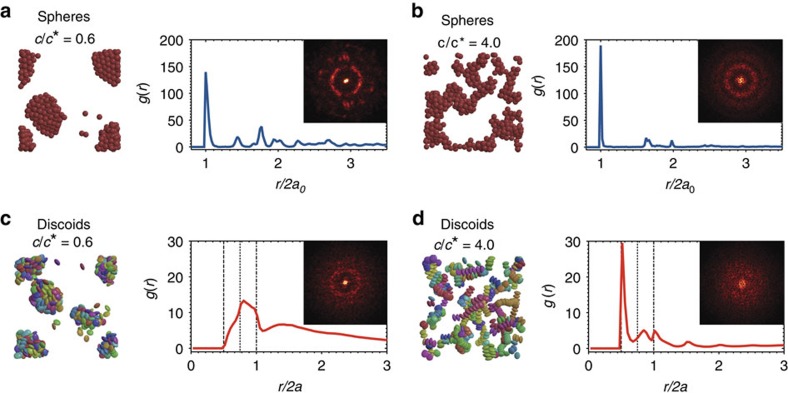
Simulations of spheres and discoids. Renderings and structural characterization from MC simulations at 40 × 10^6^ MC sweeps for (**a**) spheres at *c*/c*=0.6, (**b**) spheres at *c*/c*=4.0, (**c**) discoids at *c*/c*=0.6, and (**d**) discoids at *c*/c*=4.0. Volume fractions for (**a**-**d**) are *φ*=0.05. In **c** and **d**, discoids are coloured by their orientations. Dashed, dotted and dashed–dotted lines on the *g*(*r*) plots represent the F–F, E–F and E–E configurations. Insets in **a**–**d** show diffraction plots.

Metastable strands formed by discoids tend to progress towards disordered condensates when the attraction is weak. This order-to-disorder tendency is a result of the anisotropy of discoids, and is opposite to that of spheres, where crystalline order develops locally from initially disordered clusters (Supplementary Notes 4 and Supplementary Fig. 7). To analyse the difference, we perform a set of MC simulations of spheres with equivalent volume to the discoids (*φ*=0.05, 0.2≤*c*/c*≤4.0). Spheres interacting with depletants crystallize in a narrow region close to the coexistence boundary where liquid-gas phase separation occurs^17^. Previous experiments showed that crystalline clusters are formed in dilute suspensions of spheres^39^,^40^,^41^ (*φ*<0.10) when *U*_depl_ is moderate and short-ranged (*ξ*∼0.01), and results from our MC simulations are in agreement with these past observations. In particular, we see significant peaks in the *g*(*r*) at regular lattice spacings and local regions of high intensity in the diffraction patterns (Fig. 6a,b). On the other hand, discoids assemble into disordered condensates at moderate and low *c*/c*, with little positional or orientational order (Fig. 6c,d).

## Discussion

A colloidal system unhindered by kinetic arrest generally tends to phase separate into dense condensates and mobile particles. As attraction increases, the slowdown in kinetics could allow the persistence of metastable configurations with local energy minima^42^. Because of the increased propensity for kinetic trapping and gelation at strong attractions, the appearance of low <*z*> structures at high *c*/c* and condensed clusters at low *c*/c* is expected for both spheres and discoids^41^. The surprising observation here is that metastable orientational order appears in the initial stages of discoidal assembly across a wide range of attraction strengths. Typically, spherical systems progress from a disordered state to an ordered phase. Discoids present a different scenario because the energetically favourable F–F configuration found in strands is stabilized at high *c*/c* regardless of the final structure, discoidal assembly begins with a strong propensity for each discoid to form two F–F bonds (Supplementary Notes 5 and Supplementary Fig. 8). These initial strands are short-lived and phase separation drives them to collapse into dense clusters, unless strong attractions are used to kinetically trap strands that lengthen over time.

The evolution of orientational order occurs more slowly than clustering in discoidal assembly. In general, *f*_clust_ reaches a plateau value quickly, whereas *f*_ordered_ and <*z*> evolve much more gradually (Fig. 5). The difference in *τ*_clust_ and *τ*_ordered_ is especially apparent with weak attractions, where discoids rapidly aggregate into clusters but undergo local rearrangements over longer periods of time. Earlier simulation works on discoids showed that kinetic trapping is important in the formation of metastable structures, which occurs before the onset of large-scale structural rearrangements^43^,^44^. A perfectly helical structure can, for instance, eventually undergo hinging or stretching/compacting motion that inverts its handedness and introduces disorder^45^.

The discoidal shape of the colloids introduces a orientationally dependent interaction; orientational frustration can then result in stable structures with low coordination numbers^23^. We show that the value of <*z*> for discoids is lower than that of spheres (Supplementary Notes 6 and Supplementary Fig. 9) when very strong attractions are applied. Nevertheless, if discoids come into contact with many neighbours (particularly with weaker attractions), then the orientational specificity of the microstructure is lost. For example, a discoid that makes perpendicular contact with two F–F discoids is still in an energetically favourable state (Supplementary Fig. 10). Rapid phase separation hastens the creation of such contacts, eventually giving rise to interconnected strands. Thus, long-range orientational order is never observed with very strong attractions, even though local pairwise orientational order is broadly prevalent.

Our work demonstrates a simple, general means to generate colloidal assemblies that are both oriented and 1D by combining the effects of shape anisotropy and a quench deep into the unstable region of the phase diagram. This principle can be broadly applied to explain and predict the assembly behaviour of other types of building blocks. Incorporating anisotropy in designing microstructure and mechanical properties of materials is of general interest, for example, in colloidal gels and glasses made of ellipsoids^46^, clay platelets^26^, actin networks^29^ and granular discoids^47^ where ordering plays an important role in stress-bearing capability. Our observations are also useful for the design of polymer nanocomposites, where an understanding of the relationship between interaction anisotropy and microstructure can be harnessed to improve their elastic modulus and fracture threshold^48^,^49^.

## Methods

### Colloidal synthesis and self-assembly of PMMA discoids

Colloidal discoids are prepared by uniaxial compression of spherical particles. We synthesize the precursor monodisperse poly(methyl methacrylate) (PMMA) spherical colloids (diameter 2*a*_0_=1.64 μm±4%) stabilized by a grafted layer of poly(12-hydroxystearic acid) (PHSA) and cross-linked with ethylene glycol dimethacrylate at a 0.5 wt % ratio to the monomer^50^. The steric layer has a thickness of ∼10 nm (ref. 51). PMMA colloids are dyed with fluorescent Nile Red to allow for direct visualization with confocal microscopy. The spheres are embedded at a 2.0 wt% concentration in a hydroxy-terminated poly(dimethyl siloxane) (PDMS) (*M*_n_∼110,000, viscosity∼50,000 cSt) matrix, in which tin (II) ethylhexanoate and poly(dimethylsiloxane-co-methylhydrosiloxane) are added as a catalyst and a cross-linker, respectively. The PDMS matrix is heated to a temperature (*T*=150 °C) above the glass transition temperature, *T*_g_, of the PMMA. A uniaxial compression is applied at a pressure of 1,144 kPa (starting thickness of film=2.6 mm, final thickness=0.6 mm, strain=0.23) using two flat poly(tetrafluoroethylene) plates to generate discoids of aspect ratio, *l*=*b*/*a*=(0.46±0.09), where *a* is the radius of the major axis and *b* is the radius of the minor axis.

The thermomechanically pressed sample is allowed to cool under pressure to room temperature. A solution consisting of 0.75 wt% sodium methoxide dissolved in isopropyl alcohol and hexane is used to release the PMMA discoids from the PDMS matrix. The complete removal of PDMS is critical for self-assembly experiments. Because the degradation also chemically etches the PHSA graft copolymer from the surface of the discoids, we reconstitute the PHSA steric layer through a 72-h covalent bonding reaction catalysed by 0.24 vol% dimethylaminoethanol at *T*=80 °C (ref. 52). The reaction is performed at *T*<*T*_g_ to avoid any change to the shape of the colloids. The restabilized particles are cleaned by multiple washings with hexane and passed through a 11.0μm nylon filter to remove residue.

The value of *a* is measured from SEM images of dilute samples in which discoids lie flat on the substrate and are far apart from each other. The value of *b* is obtained from the conservation of volume of the starting sphere^27^, using the relation 
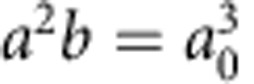
. These values are also independently verified during the image processing of confocal microscopy images. For the discoids used in this study, *a*=(1.07±0.06) μm and *b*=(0.49±0.08) μm.

Discoids are suspended via solvent transfer in a refractive index- and density-matched solvent at volume fractions varying from *φ*=0.003 to 0.04. The solvent is washed with deionized water and filtered before use, and consists of 81 vol% bromocyclohexane and 19 vol% decalin, with 1 vol% PHSA for suspension stability and 3 μM tetrabutylammonium chloride to provide charge screening. We verify the absence of sedimentation by centrifuging discoids in the solvent at 5,000 r.p.m. for 30 min, and also by suspending discoids in the solvent and monitoring changes in the volume fraction of the discoids near the coverslip over 2–3 h using confocal microscopy. From conductivity measurements^53^, the Debye length is *κ*^−1^=151 nm. The zeta potential is estimated to be *ζ*≤10 mV (ref. 35). The solvent viscosity (*η*=2.5 × 10^−3^ Pȧs) is measured with a cone-and-plate geometry on a controlled stress rheometer (TA Instruments, AR-G2).

The clean, restabilized colloidal discoids are stable in suspension for up to 4 days. Specifically, the 1D mean-squared displacement of the discoids as a function of the delay time, 
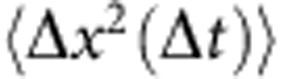
, shows that they exhibit diffusive behaviour where 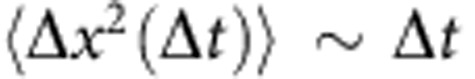 for measurements conducted at experimental wait times, *t*_w_≤192 h (Supplementary Fig. 1a). The measured orientationally averaged translational diffusivity of the dilute suspension is *D*_T_=(0.08±0.01) μm^2^ s^−1^. This value is consistent with the theoretical prediction of the orientationally averaged diffusivity calculated from parallel and perpendicular components of the Stokes–Einstein diffusivity, 
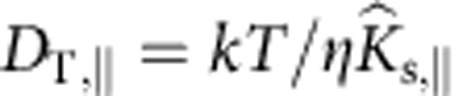
 and 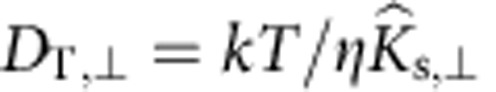, to a relative error of 15%. 
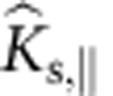
 and 
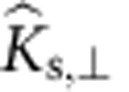
 are material constants that depend on the geometry of the discoids^54^. Here, 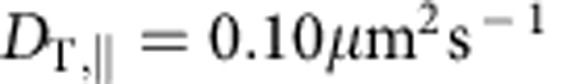 and 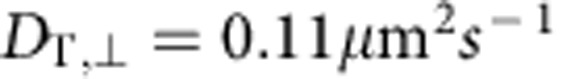 (the orientationally averaged value is plotted as a dashed line in Supplementary Fig. 1a). The self-part of the 1D time-dependent van Hove correlation function characterizes the probability distribution of single-particle displacements. Supplementary Fig. 1b shows that the discoids exhibit a Gaussian distribution in the van Hove self-correlation that is characteristic of a fluid suspension for *t*_w_≤192 h. Non-Gaussian dynamics and aggregation begin to set in thereafter, and is seen as a sharp peak at Δ*x*∼0 μm in Supplementary Fig. 1b.

Self-assembly of the discoids is induced by addition of monodisperse polystyrene (molecular weight *M*_w_=2.1 × 10^7^ g mol^−1^, radius of gyration *R*_g_=194 nm determined through static light scattering on a Wyatt Technology, DAWN EOS equipped with a 690-nm GaAs laser^55^) at various concentrations (1.1≤*c*/c*≤5.3) to the suspension. The value of c*=1.12 mg ml^−1^ for the solvent mixture used in this study is determined using the equation 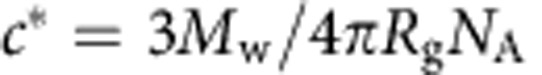 (ref. 34). The polystyrene molecules act as depleting agents that generate a long-range attraction.

### Confocal microscopy imaging

An inverted confocal laser scanning microscope (Nikon A1Rsi) equipped with a resonant scanner head and a high-speed piezo stage is used to capture the 3D structure of the self-assembly. The discoids are allowed to quiescently self-assemble in custom-built 2 ml glass vials with #1.5 (thickness=0.17 mm) glass coverslips at the bottom for a minimum of *t*_w_=120 min, and images are captured at a distance of *z*⩾10 μm above the coverslip to avoid wall interactions. The image dimensions are 63.5 × 63.5 × 30.0 μm^3^, with voxel dimensions of 124 × 124 × 124 nm^3^. The acquisition time for each image volume is ∼17 s. Image volumes are obtained from three independent samples. In addition, we collect 3D image volumes of two cases (*φ*=0.02, *c*/c*=4.0; *φ*=0.02, *c*/c*=1.7) at regular intervals from *t*_w_=0 to 120 min to study kinetics.

### Image analysis algorithm

A watershed-cut-based algorithm is used to compute the volume fraction, positions and orientations of the discoids. This method extends the identification of particle centroids based on the local brightness^56^ to arbitrary shapes. Images are segmented using watershed cuts, which divide an image into catchment basins of local minima. Pixels from which a steepest descent path leads to the same minimum are clustered together^57^. To avoid misidentification of particles in close contact (a common issue encountered with gelling or glass-forming colloids), the images are pre-flooded. That is, this algorithm requires the user to specify a depth of voxel intensity *δ* by which to fill minima, so as to eliminate spurious local minima that result in the over-identification of centroids within a particle. After pre-flooding, the eigenvectors and eigenvalues of a covariance matrix of each segment is used to compute the shape and orientation of individual particles^58^.

Using the watershed-cut method, we confirm that the aspect ratio of the discoids used in the self-assembly studies is *l*=0.50±0.01. This value is equivalent to the measurements obtained from SEM. A photopolymerized sample is used to determine the static error in the particle positions and the orientational angle, resulting in a positional uncertainty of ±20 nm in the focal plane, ±39 nm in the axial plane and an angular uncertainty of±3.0°.

### MC simulations

MC simulations are carried out using a MC plug-in to HOOMD-blue^59^,^60^,^61^ in order to investigate the self-assembly of discoids (*φ*=0.01, 0.02, 0.05, 0.10) in the presence of grand canonical implicit depletants (0.2≤*c*/c*≤4.0). A penetrable hard sphere (PHS) model is chosen for the polystyrene depletant in which depletants are treated as an ideal gas. Because the depletant-to-colloid size ratio and concentrations (*c*>c*) used in the experiments are beyond the range for which the Asakura–Oosaka model is valid^33^, many-body interactions need to be taken into account. We therefore use an implicit treatment of the depletants (ref. 32^32^). Rather than explicitly tracking the positions of the depletants, we sample a grand canonical ensemble of the PHS and place a decorrelated set of depletants in the simulation box in every move. In simulation units, the major axes and minor axes of the discoidal colloids are 1 and 0.5 respectively (*l*=*b*/*a*=0.46 in experiments), and the diameter of the depletants is 0.2 (*ξ*_a_=0.18 in experiments).

For each MC step, single colloids are allowed to rotate or translate, and the move is accepted with standard acceptance probabilities of 20%. In this system of hard particles, the repulsion is infinite if there are any overlaps between colloids and other colloids or depletants. Upon moving a colloid, we check for newly created overlaps by inserting *m* depletants uniformly and randomly in a sphere of radius *R*=*R*_col_+*R*_dep_ centred on the newly moved particle, where *R*_col_ and *R*_dep_ are the circumsphere radii of the colloid and depletant, respectively. Depletant placements that overlap with the colloid in its original position or other colloids are rejected. The number of depletants *m* is drawn from a Poisson distribution P(*m*, *λ*)=(λ^*m*^/*m*!) exp(-*λ*), where *λ*=4*π*/3(*R*^3^*ρ*_dep_) and *ρ*_dep_ is the depletant number density.

## Additional information

**How to cite this article:** Hsiao, L. C. *et al*. Metastable orientational order of colloidal discoids. *Nat. Commun.* 6:8507 doi: 10.1038/ncomms9507 (2015).

## Supplementary Material

Supplementary Information Supplementary Figures 1-10, Supplementary Notes 1-6 and Supplementary References
