# Supplementary material for: Metastable orientational order of colloidal discoids
Source: Nat Commun. 2015 Oct 7;6:8507. doi: 10.1038/ncomms9507 (PMC4633714; doi:10.1038/ncomms9507)
Supplement: Supplementary Information — Supplementary Figures 1-10, Supplementary Notes 1-6 and Supplementary References [file ncomms9507-s1.pdf]

Supplementary Figures

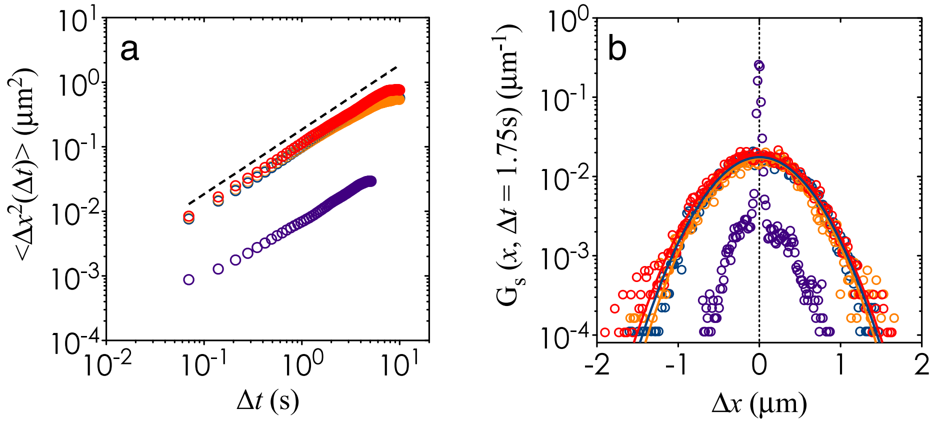

Supplementary Figure 1. Stability of colloidal discoids without added depletant. (a) The 1D mean-squared displacement as a function of lag time and (b) the single-particle van Hove correlation function for t_w = 48 hours (red), 60 hours (orange), 96 hours (blue), and 168 hours (purple). Dashed line in (a) represents the orientation-averaged Stokes Einstein translational diffusivity. Both (a) and (b) are generated from the dilute suspension shown in Fig. 1(f).

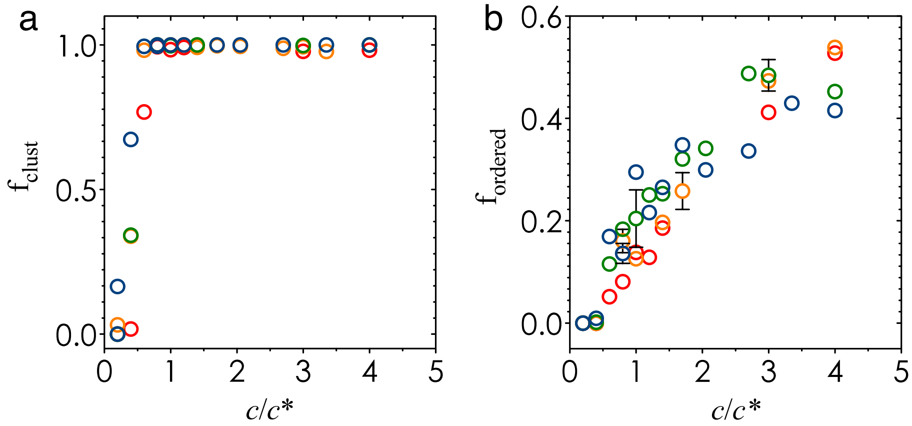

Supplementary Figure 2. Change in f_{clust} and $f_{ordered}$ as a function of *c*/*c.** Values of f_{clust} and $f_{ordered}$ plotted for ϕ = 0.01 (red), ϕ = 0.02 (orange), ϕ = 0.05 (green), and ϕ = 0.10 (blue) in MC simulations. Error bars represent standard deviation from three independent simulations.

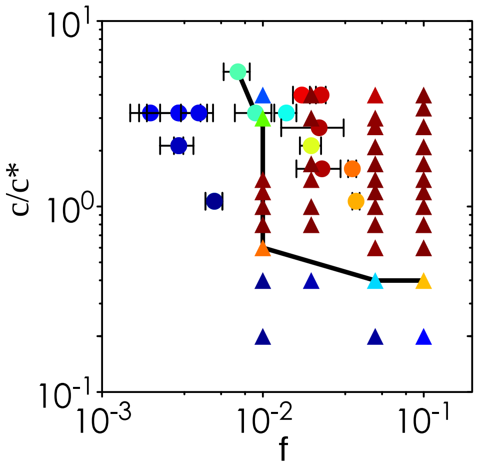

Supplementary Figure 3. Clustering phase diagram populated with experimental (circles) and simulation (triangles) state points. The color of the data points indicates the value of f_{clust}. Solid black line demarcates coexistence boundaries. Error bars represent s.e.m., n = 3.

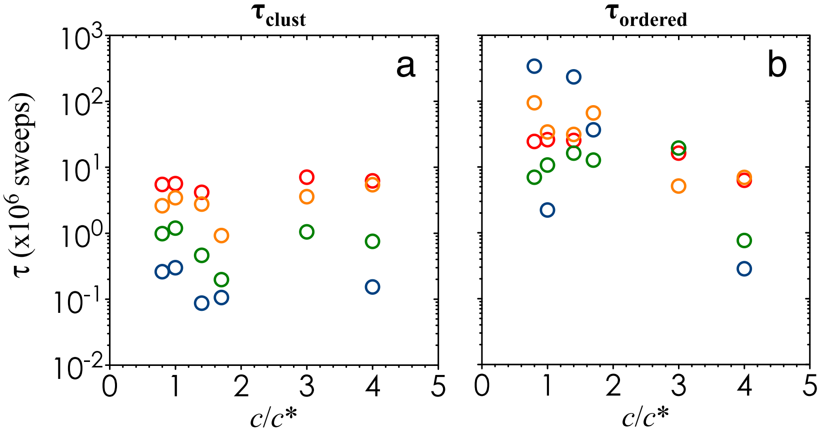

Supplementary Figure 4. Characteristic timescales for evolution of clustering and orientational order. Values of τ_{clust} and $\tau_{ordered}$ plotted for $\phi = 0.01$ (red), $\phi = 0.02$ (orange), $\phi = 0.05$ (green), and $\phi = 0.10$ (blue) in MC simulations.

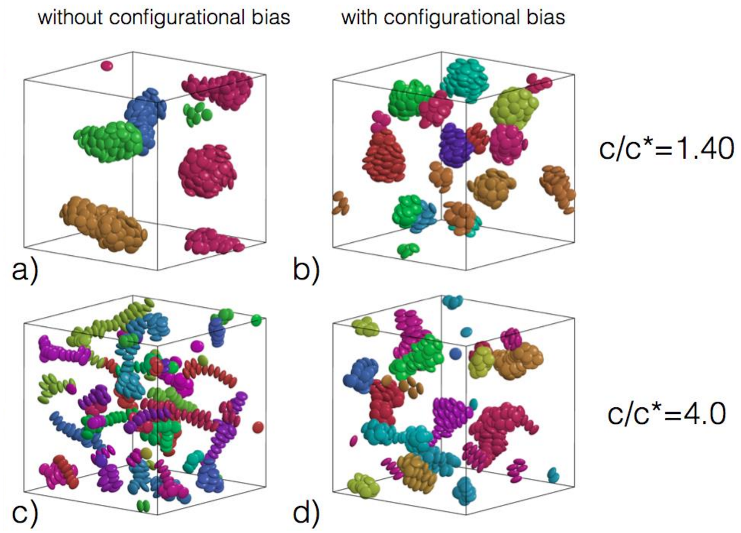

Supplementary Figure 5. Different dynamics lead to different non-equilibrium configurations at high *c*/*c.** Shown are final configurations from MC simulations after (a) 41×10^6, (b) 315.2×10^6, (c) 28×10^6, (d) 72×10^6 MC sweeps, for two different depletant fugacities $c/c^* = 1.4$ (a,b), and for $c/c^* = 4.0$ (c,d). The left column shows configurations obtained without configurational bias ($n_{\mathrm{trial}} = 0$), the configurations in the right columns were obtained with (b) $n_{\mathrm{trial}} = 100$ and (d) $n_{\mathrm{trial}} = 750$.

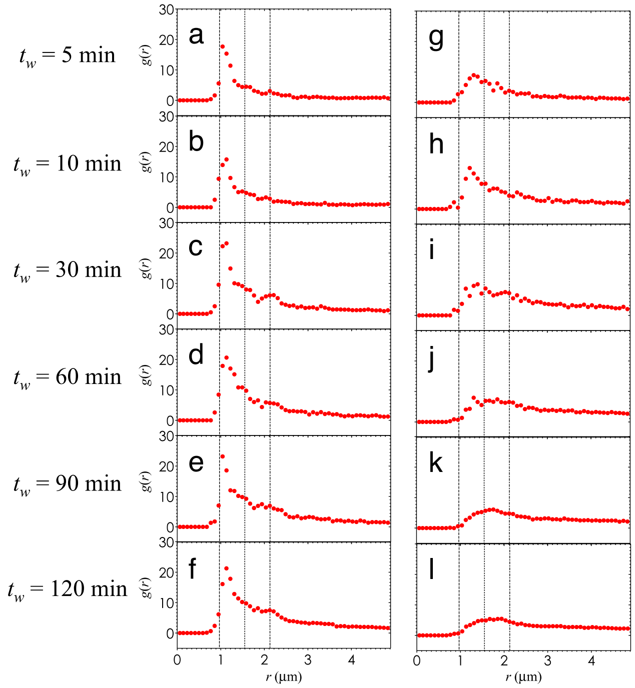

Supplementary Figure 6. Radial distribution function of self-assembled structures as a function of time. (a-f) c/c^* = 4.0, ϕ = 0.02; (g-l) c/c^* = 1.7, ϕ = 0.02. Dashed, dashed-dotted, and dotted lines represent the F-F, E-F, and E-E configurations.

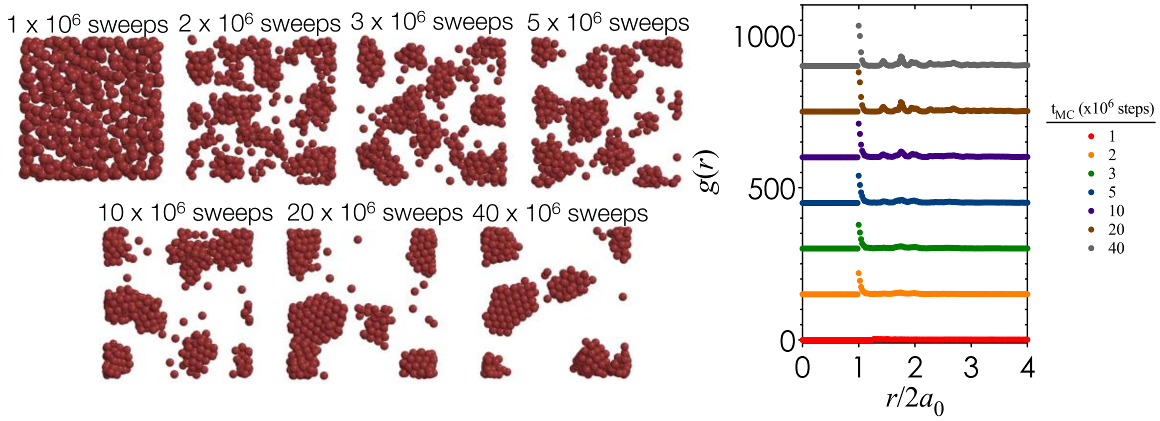

Supplementary Figure 7. Development of crystallinity in simulations of spheres at ϕ = 0.05, *c*/*c = 0.6.** Renderings of simulated structures from $t_{\mathrm{MC}} = 1 \times 10^6$ to 40×10^6 sweeps are given. The $g(r)$ plots for each corresponding time step are also shown. Legend for time sweeps are given in the inset.

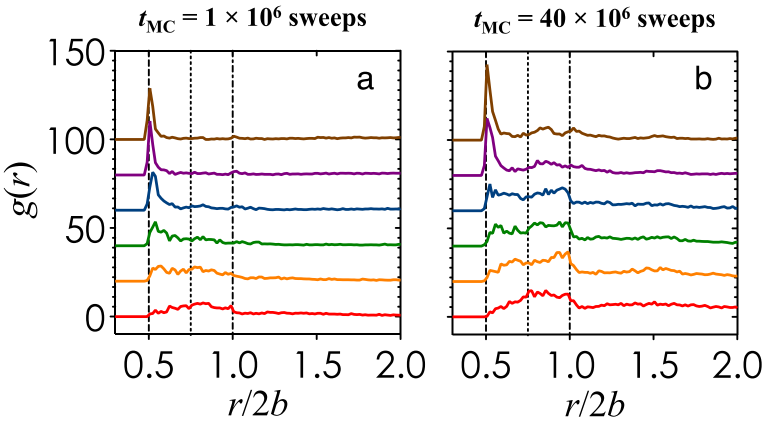

Supplementary Figure 8. Characterization of initial and final discoidal assembly structure from simulations. The $g(r)$ plots are shown at (a) $t_{MC} = 1 \times 10^6$ sweeps and (b) $t_{MC} = 40 \times 10^6$ sweeps, for $c/c^* = 0.6$ (red), $c/c^* = 1.0$ (orange), $c/c^* = 1.4$ (green), $c/c^* = 2.1$ (blue), $c/c^* = 3.4$ (purple), and $c/c^* = 4.0$ (brown). Dashed, dashed-dotted, and dotted lines represent the F-F, E-F, and E-E configurations. The $g(r)$ plots are offset for clarity.

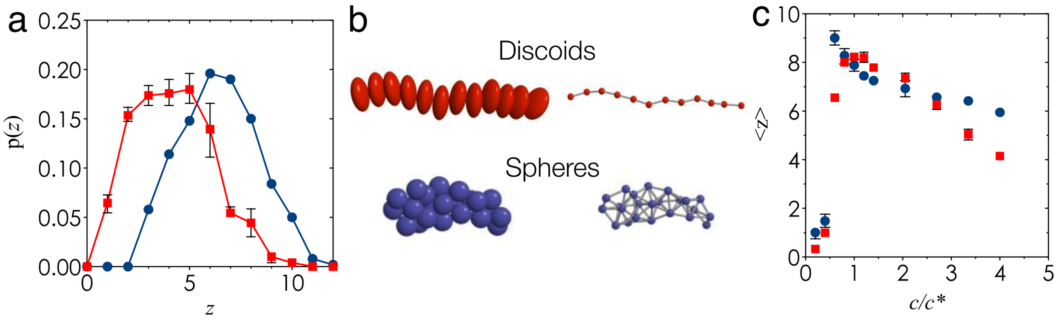

Supplementary Figure 9. Contact number distributions of discoids and spheres. (e) Contact number distribution of discoids (red) versus spheres (blue) at 40×10^6 MC sweeps, both at ϕ = 0.05 and *c/c** = 4.0. (f) Representative snapshots and bond diagrams of strands formed by discoids and tetrahelices formed by spheres (*c/c** = 4.0, ϕ = 0.05). (g) <*z*> as a function of *c/c** at ϕ = 0.05 for discoids (red) and spheres (blue). Error bars represent s.e.m., *n* = 3.

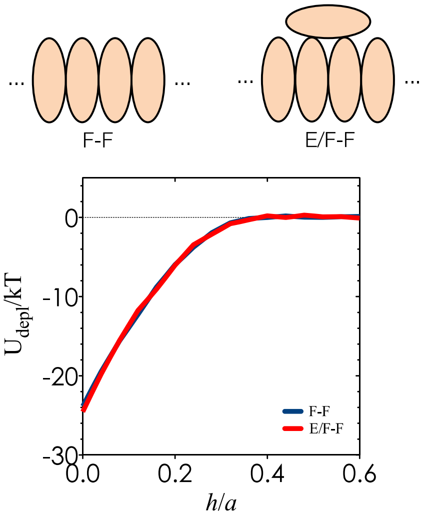

Supplementary Figure 10. Pairwise depletion interaction of E/F-F and F-F contacts. Schematic of the two different configurations, along with the plot of U_{depl} at c/c^{*} = 1.5 for F-F contacts (blue) and E/F-F contacts (red) are shown.

Supplementary Notes

Supplementary Note 1: Analytic discoid-discoid overlap check in MC simulations

The detection of discoid overlaps and the computation of the distance of closest approach is used in the MC simulations[1, 2]. The surface of a discoid of arbitrary orientation can be written as $Ax^2 + By^2 + Cz^2 + Dxy + Eyz + Fxz + Gx + Hy + Jz + K = 0$. This can be written as a 4 × 4 matrix equation,

$$\begin{pmatrix} x & y & z & 1 \end{pmatrix} \frac{1}{2} \begin{pmatrix} 2A & D & F & G \\ D & 2B & E & H \\ F & E & 2C & J \\ G & H & J & 2K \end{pmatrix} \begin{pmatrix} x \\ y \\ z \\ 1 \end{pmatrix} = 0$$

We can choose the origin to be at the center of discoid A. Discoid B is translated a distance $\{x_0; y_0; z_0\}$, by first computing the appropriate matrix **M** about its center and translating it by multiplying by **T** defined as

$$\mathbf{T} = \begin{pmatrix} 1 & 0 & 0 & 0 \\ 0 & 1 & 0 & 0 \\ 0 & 0 & 1 & 0 \\ -x_0 & -y_0 & -z_0 & 1 \end{pmatrix}$$

Matrices for discoids A and B are thus $\mathbf{A} = \mathbf{M}_A$ and $\mathbf{B} = \mathbf{T}^T\mathbf{M_B}\mathbf{T}$. To detect separation between discoids, we invoke the theorem proved by Wang et al.[3] that the two discoids overlap if and only if the *f*, defined as

$$f(\lambda) = \det(\lambda \mathbf{A} + \mathbf{B}) = 0$$

has two positive roots. The number of roots of this equation is checked using Sturm's theorem. The values in the Sturm chain can be directly calculated from the coefficients of $f(\lambda)$ - a direct implementation was numerically unstable due to round-off errors. However, by exploiting the identity

$$\lambda^4 \det(A + \lambda^{-1} B) = \det(\lambda A + B)$$

we are able to compute the Sturm chain for both determinants efficiently and use the chain with the smallest round-of errors to detect overlaps. The current implementation in HPMC, the hard-particle Monte-Carlo plugin we developed for HOOMD-blue, passes a stress test of over 100 pathological configurations.

Supplementary Note 2: Dependence of the morphology on the dynamics at high values of *c*/*c**

At $c/c^* \geq 3.0$ we observe the formation of short, long-lived metastable strands, which slowly coarsen into more disordered, multi-stranded and linked configurations. In MC simulation, we can tune the speed at which this relaxation occurs using a configurational bias scheme[3] that enhances the collective diffusivity of self-assembled strands, as well as that of single colloids in the depletant bath. By reinserting overlapping depletants n_{trial} times at the new position of the moved colloid into the old position – and vice versa, to guarantee reversibility of the dynamics – we compute the bias weight that multiplies the acceptance probability of the single particle moves. We fix the average

acceptance rate (at 20%), so that higher numbers of n_{trial} lead to larger step sizes. As a result, the collective relaxation of strands is promoted.

Supplementary Fig. 5(a, b) show that for moderate values of c/c^* = 1.4, no significant difference in morphology is observed. However, the metastable strand-like configurations at high c/c^* = 4.0 are more long-lived when collective relaxation is suppressed (n_{trial} = 0, Supplementary Fig. 5(c)), whereas amorphous clusters are more dominant with configurational bias moves (n_{trial} > 0, Supplementary Fig. 5(d)). Such dependence on the dynamics strongly supports the out-of-equilibrium character of the strand configuration.

Supplementary Note 3: Time-dependent radial distribution functions from experiments of discoids

In Supplementary Fig. 6 (a-f), the $g(r)$ for the sample with the highest degree of orientational order (f_{ordered} = 0.36 ± 0.07) exhibits a sharp first peak at $r/2a$ = 0.5 at all t_w, corresponding to the F-F configuration. The intensity of the peak increases as a function of t_w and shows that the subpopulation of particles in the F-F configuration increases. For samples with low orientational order (f_{ordered} = 0.07 ± 0.03), Supplementary Fig. 6(g-l) show that the peaks of the $g(r)$ at all t_w are much lower, and a broad peak centered at $r/2a$ = 0.75 is fully established by t_w = 90 min. Particles in this assembly exist primarily in the E-F and E-E configurations at t_w = 120 min.

Supplementary Note 4: Crystalline clusters in simulation of spheres

Supplementary Fig. 7 illustrates the development of crystalline clusters with volumetrically-equivalent spheres (ϕ = 0.05) at c/c^* = 0.6. Renderings from simulations show that the spheres are in a randomly thermalized state initially. As the simulation time progresses to 40×10^6 sweeps, the spheres assemble into clusters with increasingly prominent positional order. This increase in crystallinity is quantified by the $g(r)$ plot, where the primary peak at $r = 2a_0$ increases in intensity over time and secondary peaks begin to appear. (The data are offset to provide clarity).

Supplementary Note 5: Time-dependent radial distribution functions from simulations of discoids

Similar to the time-dependent $g(r)$ from experiments, the simulations show that metastable oriented strands are formed initially at short times, independent of the final structure at long times. In particular, the kinetics at c/c^* = 2.1 (blue lines) are sufficiently slow to allow the initial metastable states ($t_{\mathrm{MC}} = 1 \times 10^6$ sweeps) to be observed as a sharp peak at in the $g(r)$ plot at $r/2b$ = 0.5 (Supplementary Fig. 8(a)). At long times ($t_{\mathrm{MC}} = 40 \times 10^6$ sweeps), phase separation dominates the metastable states and results in a dense cluster with no orientational preference (Supplementary Fig. 8(b)). These time-dependent $g(r)$ plots provide quantitative evidence that the initial metastable states are short-lived unless deep quenches are used to generate kinetic trapped strands that can grow over time.

Supplementary Note 6: Contact number distributions in isotropic and discoidal assembly

As the depletant concentration is raised, we showed that discoids begin to assemble into long strands (Fig. 3). While spheres and discoids both assemble into clusters of linear strands, the contact number of the two types of colloids are distinct. Spherical colloids pack closely, with a mean value of $\langle z \rangle = 6.0 \pm 0.2$ (Supplementary Fig. 9(a)). On the other hand, discoids assemble into strands with a lower value of $\langle z \rangle = 4.2 \pm 0.3$. Close-up snapshots of the rendered structures show that spheres assemble in a tetrahelical motif similar to that seen with short-range attractions, while the discoids form strands of lower contact number (Supplementary Fig. 9(b)). The difference in these non-equilibrium structures is most distinguishable at high c/c^* (Supplementary Fig. 9(c)). The lower value of $\langle z \rangle$ seen with discoids is likely due to the anisotropic potential energy of the energetically favorable F-F configurations.

Supplementary References

1. Wang W, Wang J, Kim M-S. An algebraic condition for the separation of two ellipsoids. *Computer Aided Geometric Design* **18**, 531-539 (2001).
2. Alfano S, Greer ML. Determining if two solid ellipsoids intersect. *Journal of Guidance Control and Dynamics* **26**, 106-110 (2003).
3. Bolhuis P, Frenkel D. Numerical Study of the Phase-Diagram of a Mixture of Spherical and Rodlike Colloids. *Journal of Chemical Physics* **101**, 9869-9875 (1994).
